# Pennsylvania Hospital—Surgical Wards—Service of Dr. Norris

**Published:** 1842-06-11

**Authors:** E. Hartshorne

**Affiliations:** Resident Surgeon


### *Pennsylvania Hospital—Surgical Wards—Service of Dr. Norris.*

By E. Hartshorne, M. D., Resident Surgeon.

*Fractured Thigh treated three weeks at sea without the production of callus—Accidental refracture during convalescence—Re-union quite firm within twenty days after the second accident.*—Joseph P., aged 45, temperate, healthy, and robust in frame, was admitted February 17th, with oblique fracture of the right thigh bone in its middle third, of twenty-one days standing. The man had met with the accident in the island of Porto Rico, where the fracture was reduced within half an hour after its occurrence, and the limb dressed with the long splint of Desault. This apparatus was continued, under the management of the master of the vessel, during a three weeks' voyage, until the patient arrived in this port and entered the Hospital. At that time ligamentous union alone had taken place, and without any provisional callus ; the foot was somewhat everted, and the lower fragment was drawn upwards and inwards, overlapping the upper, as usual in recent cases of this kind, so as to produce one inch of shortening. The limb was immediately extended with moderate force, and so retained in Physick's modification of Desault's apparatus. Care of course was taken to preserve the foot in the proper position, from which it had previously been allowed to fall by its own weight; and the thigh itself, especially around the seat of fracture, was subjected, by means of a compress and an additional band around the splints at that part, to firm compression. The patient was allowed a nutritious diet, with a pint of porter daily. Pressure and rest were still better secured at the end of the second week by the addition of paste-board splints fitted to the thigh while wet, and tightly bandaged with a roller enveloping the whole extremity.

An unusually large amount of callus was soon thrown out, and consolidation appeared to have become quite firm by the twenty-sixth day of his treatment on land. The splints of Desault were removed on the thirtieth day, the paste-board and bandage being still employed.

In the course of the second or third night after the removal of the long splints, the man fractured the callus by starting, while asleep, with his right foot entangled in the sheet. He was roused by the pain and snapping of the bone, and at once perceived that motion and pain had re-appeared in the breach of continuity, while he was again deprived of the use of the limb.

The splints were re-applied the next morning, and were not taken off for twenty days. By this time union, which advanced rapidly in the new deposite, had been again completed. The reparative action went on the second time with so much celerity that ere four days had elapsed it was not easy to detect any motion at the seat of injury ; nor at the end of two weeks did any exist.

He was allowed to leave his bed after some forty-five days confinement. Discharged May 7th cured, with the affected limb about three-fourths of an inch shorter than the other.

*Fractured tibia and fibula of twenty-three days standing.—Retarded union.*—John R., aged 22, temperate, strong, and healthy, fractured the bones of his right leg transversely, about five inches above the ankle-joint. The accident occurred at sea three weeks and two days before his entrance into these wards. The fracture had been reduced soon after the accident, and a rude fracture-box, lined with raw cotton, had been employed while he was aboard ship, to retain the parts at rest in tolerably good position. The patient was admitted into the Hospital March 23d. Very slight union had at that period taken place, the foot was partially inverted, and slight internal angular deformity existed at the seat of fracture.

The fragments having been restored as near as possible to their right position, paste-board, moulded to the leg while wet, was made to produce firm and uniform compression by means of a roller carefully applied as usual to the whole extremity below the knee. The limb thus dressed was confined at rest on a well stuffed pillow in a fracture box, and a good diet with half a pint of porter daily was prescribed.

In fifteen days consolidation had become firm; and in twenty the man was allowed to walk about, the limb being still supported by the paste-board and roller. Discharged May 5th cured, with a considerable amount of callus, but no material displacement of the fragments.

*Remarks.*—These cases are chiefly interesting as examples of retarded union, arising most probably from want of sufficient rest. The apparatus intended to secure the fragments against the effects of motion were, in both instances, but poorly adapted to the purpose even on shore, where complete repose is so much more easily obtained, while they had been rarely re-adjusted. The man, moreover, suffered under the disadvantages of a bad diet, and all the other discomforts of a sailor's berth at sea.

In both individuals cures were obtained within a reasonably short period, and without appreciable deformity, (except the slight shortening not overcome in the case of fractured femur,) merely by the ordinary treatment for similar lesions in the recent stage, combined with moderate compression of the part involved. The first case furnishes also an interesting illustration of the celerity with which union is re-established in a provisional callus fractured before the process of consolidation has been entirely confirmed. As might have been expected under the circumstances, no distinct crepitation could be perceived in either case when first examined, or between the new surfaces resulting from refracture of the thigh.

